# Surgical Delusions and Follies

**Published:** 1884-12

**Authors:** 


SURGICAL DELUSIONS AND FOLLIES. A review of the Address in surgery for 1884 of the Medical Society of the State of Pensylvania. By John B. Roberts, M. D, Professor of Anatomy and Surgery in the Philadelphia Polyclinic, surgeon to St. Mary's Hospital. P. Blackiston, Son & Co., Philadelphia 1884.

In the condensation of "Surgical Delusions" into 19 distinct propositions, six are found untenable, while thirteen points are well made out; and to render our meaning clear, the enumeration for the former are, 1, 2, 3, 5, 7 and 15; while, in the regular order, the latter are, 4, 6, 8, 9, 10, 11, 12, 13, 14, 16, 17, 18, 19.

Under the head of "Surgical Follies," there are thirteen items, of which four do not stand the test of practicability, while nine are properly sustained, the former having numbers 1, 3, 6 and 10, and the latter numbers 2, 4, 5, 7, 8, 9, 11, 12 and 13.

If numbers are affixed to the two separate divisions, our qualification of each will be comprehended and appreciated, without further details as to "their right to be classed as surgical facts."

This is a handsome little book, well written, and will be read with profit by every one.

